# *Aquaporin 3* Promoter Polymorphisms Reduce Gene Expression and Associate Altered Autophagy‐Related Gene Signatures With Atopic Dermatitis Susceptibility

**DOI:** 10.1111/exd.70358

**Published:** 2026-09-15

**Authors:** Yi Ying Eliza Lim, Yang Yie Sio, Terence Yin Weng Lam, Yee‐How Say, Kavita Reginald, Fook Tim Chew

**Affiliations:** ^1^ Department of Biological Sciences, Faculty of Science National University of Singapore Singapore Singapore; ^2^ Department of Biomedical Science, Faculty of Science Universiti Tunku Abdul Rahman (UTAR) Kampar Campus Kampar Malaysia; ^3^ Department of Biomedical Sciences, Sir Jeffrey Cheah Sunway Medical School, Faculty of Medical and Life Sciences Sunway University Subang Jaya Malaysia

**Keywords:** aquaporin 3, atopic dermatitis, autophagy, genome‐wide association studies, inflammation

## Abstract

Genome‐wide association studies (GWAS) have uncovered multiple loci associated with atopic dermatitis (AD), although mechanistic understanding remains limited. Here, we examine a risk variant in the *aquaporin 3* (*AQP3*) promoter and propose a mechanistic link between genotype to altered autophagy, skin barrier integrity, and wound healing in AD. A GWAS involving 1261 AD cases and 4062 controls of Chinese ancestry from the Singapore–Malaysia cohort was performed. Linkage disequilibrium (LD) analysis and cis‐eQTL datasets were used to assess regulatory associations. Functional effects of promoter variants were validated using in vitro luciferase assays and ex vivo transcriptomic profiling. In vitro experiments in HaCaT keratinocytes investigated the effect of siRNA‐mediated *AQP3* knockdown on autophagy, skin barrier integrity, and wound closure. A discovery GWAS identified significant association between AD and SNP rs12555686 (*p* = 1.67 × 10^−11^, OR = 1.89), located in the promoter of *AQP3*. Two additional SNPs in strong LD (rs2231225, rs3758279) formed a major haplotype associated with reduced *AQP3* expression. Reduced *AQP3* expression was associated with decreased autophagy markers (*Beclin1*, *ATG5*, *ATG12*, *GABARAPL2, LAMP2, ATG4C*), increased senescence markers (*p21*, *GADD45A*, *SERPINE1*), and elevated inflammatory cytokines (*IL8*, *IL1B*, *TNF*). Lastly, *AQP3* knockdown in HaCaT keratinocytes reduced steady‐state LC3B‐II/LC3B‐I ratio, reduced occludin expression, and delayed wound healing in vitro. Promoter variants in *AQP3* link genetic susceptibility to gene signatures consistent with altered autophagy, cellular senescence, and inflammation in AD. These findings highlight *AQP3* as a potential modulator of both epithelial and immune‐related mechanisms in AD pathogenesis.

## 1 Introduction

Atopic dermatitis (AD) is a chronic inflammatory skin disorder characterized by epidermal barrier dysfunction, heightened immune responses, and recurrent episodes of pruritic eczema. Although genome‐wide association studies (GWAS) have uncovered several risk loci for AD, many map to non‐coding regions with unclear regulatory functions [1]. Understanding how such variants influence disease‐relevant pathways is critical for advancing targeted therapeutic approaches.

Aquaporin 3 (AQP3) is a channel protein highly expressed in the skin. It facilitates the transport of water, glycerol, and small solutes across the plasma membrane and plays a critical role in skin hydration, elasticity, and barrier repair. Mouse models have demonstrated that loss of AQP3 impairs cutaneous water content and delays barrier recovery following injury, highlighting its importance in maintaining epidermal homeostasis [2]. Beyond its classical transport functions, emerging studies reveal that AQP3 can also act as a molecular scaffold that coordinates intracellular signalling pathways. Of particular interest is its ability to modulate autophagy [3, 4]. AQP3 interacts with Beclin 1 and can disrupt its association with death‐effector domain‐containing protein (DEDD), thereby facilitating the initiation of autophagosome formation [5]. These non‐canonical roles implicate AQP3 in diverse cellular stress responses, including those governing inflammation, oxidative stress, and cellular senescence. Although *AQP3* expression has been reported to be dynamic and responsive to environmental cues, such as how prior reports have shown that ultraviolet (UV) radiation can alter *AQP3* expression through distinct signalling pathways, it is unclear how genetic variants in the *AQP3* regulatory region influence its transcriptional regulation in the context of disease.

In this study, we identify a promoter variant in *AQP3* (rs12555686) through GWAS in a Singapore–Malaysia Chinese cohort and demonstrate its functional role on transcriptional regulation. By integrating eQTL datasets, luciferase reporter assays, and transcriptomic profiling of peripheral blood mononuclear cells (PBMCs), we demonstrate that regulatory haplotypes at the *AQP3* locus significantly modulate gene expression and may contribute to disease progression through altered autophagy‐related and senescence‐associated gene signatures. In parallel, siRNA‐mediated genetic knockdown of *AQP3* reveals reduced steady‐state LC3B‐II/LC3B‐I ratio, reduced occludin expression, and delayed wound healing in HaCaT keratinocytes. These findings are consistent with a model in which promoter variants regulating *AQP3* expression may in turn modulate autophagy and regenerative capacity in AD pathogenesis.

## 2 Materials and Methods

### 2.1 Participant Recruitment and Population Ascertainment

Eligible subjects must be of Chinese ethnicity, have abstained from antihistamines for at least 3 days prior to the study and at least 18 years of age, with a mean age of 21.87 (SD = 5.27). A skin prick test (SPT) to house dust mite (HDM) was conducted for all participants, determined to be sufficient in diagnosing atopy status according to findings by Andiappan et al., with high sensitivity and specificity [6, 7]. AD was defined in participants as having AD symptoms (recurrent flexural itch lasting six months or longer) with a positive atopic status [8]. Controls were those without AD symptoms.

### 2.2 Genotyping and Genome‐Wide Association Studies

Participants provided mouthwash samples from which genomic DNA was extracted using the Axygen AxyPrep Multisource Genomic Miniprep DNA kit. Genotyping was then performed using five GWAS arrays (Infinium OmniZhongHua‐8 v1.3 BeadChip platform, Illumina HumanHap 550 k BeadChip version 3, InfiniumOmni2‐5Exome, Infinium Global Screening Array, and Infinium OmniZhonghua‐8 v1.4 BeadChip platform). Data from the 1000 Genomes Project Phase III Beijing Han Chinese (CHB) database was used to conduct haplotype phasing and imputation separately for each array using the IMPUTE v2.0 program. These have shown to have high minor allele frequencies (MAF) with the Singapore Chinese [9, 10]. Results from all 5 arrays were integrated and subjected to quality control. Individuals with call rates below 90%, discrepancies in gender information, and duplicates or related samples were excluded. Additionally, SNPs with call rates below 90%, indels, variants with imputation scores below 0.3, duplicates, monomorphic SNPs, and variants failing the Hardy–Weinberg equilibrium (HWE) threshold (*p* < 0.001) were excluded, following recommended GWAS quality control (QC) criteria [11, 12]. This has resulted in a dataset of 4 966 935 merged SNPs. As previously confirmed by principal component analysis (PCA), no apparent stratification across platforms was observed, and the genetic architecture was comparable to the CHB population from the 1000 genomes database [13]. Altogether, we had 5323 unique participants (1261 AD cases and 4062 controls) for subsequent analysis of SNP‐disease associations.

GWAS was performed with adjustments for age, gender, and 10 principal components (PCs). Genetic variants were limited to those with minor allele frequencies (MAF) ≥ 5% and those with *p* < 5 × 10^−8^ were considered to be associated with disease. Manhattan and quantile‐quantile plots were generated using the R program v3.6.2 qqman package. Odds ratio (OR) was calculated using PLINK v1.09. Haploview version 4.2 was used to compute r^2^ values, visualize linkage disequilibrium (LD) patterns, and determine haplotype frequencies for gene constructs [14].

### 2.3 Reporter Gene Assay

DNA fragments of the top two most frequently occurring haplotypes containing *AQP3* promoter SNPs spanning from 33 449 596 to 33 447 530 base pairs (Chr9, GRCh38/hg38) were inserted into promoter‐less pGL4.10 plasmids and transfected into human embryonic kidney 293 T (HEK293T) cells (ATCC, Manassas, VA) using Lipofectamine 2000 reagent (Invitrogen, Singapore). Control plasmid pGL4.74 containing the renilla luciferase reporter gene was co‐transfected to normalize for transfection efficiency. Cells were harvested at 24‐ or 48‐h post‐transfection, and luciferase activity was measured using Promega's GloMax Discover Microplate Reader and Dual Luciferase Reporter Assay Kit (Promega, Singapore) as per the manufacturer's protocol. Firefly luciferase activities were normalized to the renilla luciferase activity to obtain relative luciferase units. Transfection with the empty vector pGL4.10 [luc2] served as a control, and all experiments were repeated three times.

### 2.4 Gene Expression Association Analysis

Total mRNA expression was quantified from PBMCs isolated from blood samples using the E.Z.N.A. Total RNA kit (Omega Bio‐tek Inc.). mRNA enrichment was performed with oligo(dT) beads, and sequencing libraries were prepared using the NEBNext Ultra RNA Library Prep Kit, followed by sequencing on an Illumina NovaSeq 6000 platform. Raw reads were aligned to the human reference genome (GRCh37/hg19) with TopHat v2.1.1, and transcript abundance was estimated as fragments per kilobase of transcript per million mapped reads (FPKM) using Cufflinks v2.2.1. Raw counts were normalized for sequencing depth and gene length, and FPKM values underwent quantile normalization.

Transcriptomic analysis was conducted to investigate the association between *AQP3* expression levels and genes involved in autophagy and cellular senescence. RNA‐seq expression data were analysed in R (version 4.3.0) using the packages ggplot2, ggpubr, dplyr, readr, and tidyr. Samples were stratified into “High” and “Low” *AQP3* expression groups based on a median or quartile split. For each gene of interest, expression distributions were visualized using box and whisker plots, excluding outliers (outlier. shape = NA) and overlaying individual data points with jitter for clarity. Senescence‐associated transcripts and autophagy‐related markers were pre‐specified based on their established biological roles identified through literature review prior to the transcriptomic analyses [15, 16]. Senescence‐associated transcripts included *p21 (CDKN1A)*, *IL1B*, *IL8*, *TNF*, *GADD45A*, and *SERPINE1*, whereas autophagy‐related markers included *Beclin 1*, *ATG5*, *ATG12*, *GABARAPL2*, *LAMP2*, and *ATG4C*. Statistical differences between the *AQP3*‐high and ‐low groups were assessed using two‐tailed Mann–Whitney U tests, and the Benjamini‐Hochberg procedure was applied to calculate the false discovery rate (FDR). SNP‐gene associations were also investigated in multi‐tissues using publicly available databases eQTLGen Phase II (accessible at: https://www.eqtlgen.org/cis‐eqtls.html) and GTExPortal version 10 (accessible at: https://gtexportal.org/).

### 2.5 Cell Culture

HaCaT keratinocytes (Cytion, #300493) were cultured in Dulbecco's Modified Eagle Medium (DMEM; HyClone) and supplemented with 10% heat‐inactivated foetal bovine serum (FBS; HyClone, #SV30160.03HI) maintained at 37°C in a humidified incubator with 5% CO_2_. For siRNA‐mediated *AQP3* knockdown, cells were seeded at a density of 0.1 × 10^6^ cells per well in 12‐well plates (1 mL/well) and incubated overnight to reach 50% confluency. The next day, cells were washed with PBS and replenished with fresh DMEM containing 10% FBS before siAQP3 knockdown using Lipofectamine RNAiMAX (Invitrogen, 13 778 075) and 10 nM AQP3 siRNA (Santa Cruz Biotechnology, sc‐29 713). After 24 h, cells were washed with PBS, switched to serum‐free DMEM and stimulated with 10 ng/mL TNFα (MedChemExpress, HY‐P70426A) and/or 20 ng/mL IFNγ (MedChemExpress, HY‐P7025) for 24 h. All experiments were repeated three times. *AQP3* siRNA, TNFα and IFNγ did not significantly affect HaCaT cell viability when treated individually or in combination with the specified doses.

### 2.6 Western Blot and Immunocytochemistry

HaCaT keratinocytes were harvested using RIPA lysis buffer containing protease and phosphatase inhibitors (MedChemExpress, HY‐K0013). Western blots were carried out using anti‐AQP3 (Abcam, 13 003–2‐AP), anti‐LC3B (Cell Signalling Technology, #3868), anti‐SQSTM1/p62 (Cell Signalling Technology, #5114), anti‐Occludin (Proteintech, 13 409–1‐AP), anti‐Claudin 1 (Proteintech, 13 050–1‐AP), and anti‐β‐actin (Santa Cruz Biotechnology, sc‐47 778). Immunocytochemistry was carried out using anti‐Occludin (Proteintech, 13 409–1‐AP), anti‐Claudin 1 (Proteintech, 13 050–1‐AP), and DAPI (Thermo Fisher Scientific, D1306). Densitometric quantifications and statistical analyses were performed using Prism (Graphpad). Comparisons between groups were done using two‐way ANOVA with Tukey's post hoc test.

### 2.7 Scratch Wound Assay

HaCaT keratinocytes were seeded at a density of 0.2 × 10^6^ cells per well in 6‐well plates (2 mL/well) and incubated overnight to reach 50% confluency. After 24 h, cells were washed with PBS and replenished with fresh DMEM containing 10% FBS before siRNA‐mediated *AQP3* knockdown using Lipofectamine RNAiMAX (Invitrogen, 13 778 075) and 10 nM *AQP3* siRNA (Santa Cruz Biotechnology, sc‐29 713). The next day, confluent HaCaT keratinocytes were scratched using a SPLScar Scraper (SPL Life Sciences), washed twice with PBS and switched to serum‐free DMEM. Wound closure was measured after 24 h and quantified using ImageJ. Statistical analysis was performed using Prism (Graphpad) and comparison between groups was conducted using two‐tailed Student's *t*‐test. The experiment was repeated three times.

## 3 Results

### 3.1 *AQP3* Promoter Variant rs12555686 Is Associated With AD and Reduced Gene Expression

**FIGURE 1:**
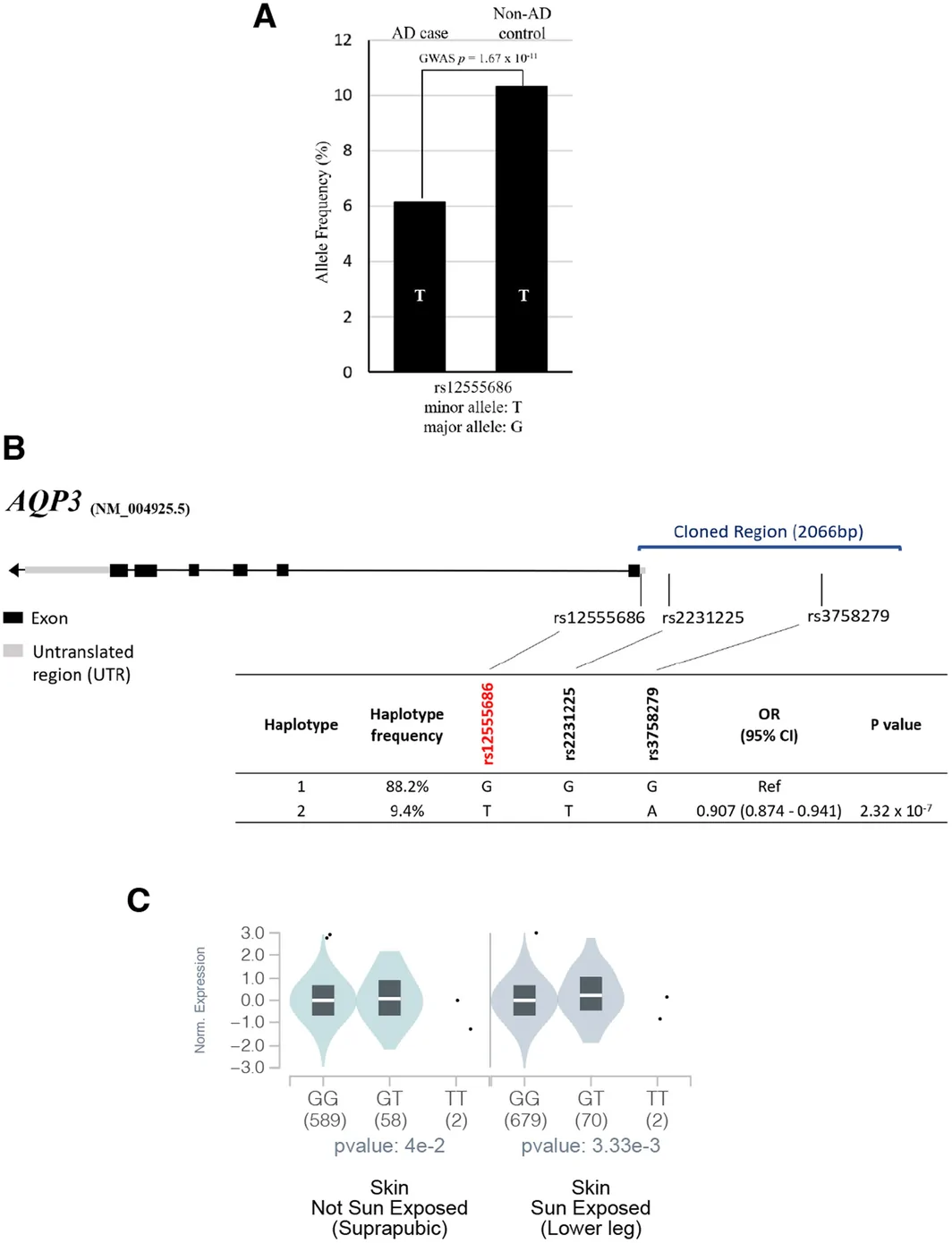
Single nucleotide polymorphism (SNP) rs12555686 association with AD cases and location in gene. (A) Percentage frequency of SNP rs12555686 major G allele and minor T allele in AD cases and controls. Major allele G is significantly associated with AD cases (*p* = 1.67 × 10^−11^). (B) *Aquaporin 3* (*AQP3*) gene transcript variant NM_004925.5 gene structure with the relative positions of atopic dermatitis‐associated SNP rs12555686 (red font) and its two‐linkage disequilibrium (r^2^ > 0.75) SNPs rs2231225 and rs3758279 in the promoter region. Haplotype analysis shows haplotype 1 (88.2% frequency) consisting of major alleles of all three SNPs to be associated with AD cases (*p* = 2.32 × 10^−7^). (C) EQTL analysis of *AQP3* SNP rs12555686 in skin samples from GTEx Portal v10. Sample sizes indicated in parentheses and nominal regression *p* values indicated below.

A discovery GWAS was performed on the SMCGES cohort comprising 1261 AD cases and 4062 controls. The genotyping data passed all QC, including HWE filtering, and showed minimal evidence of genomic inflation (genomic inflation factor, λ = 1.06406), as demonstrated by the quantile–quantile (QQ) plot (Figure S2). A significant GWAS‐level of association was identified at chromosome 9p13.3, with the lead SNP rs12555686 (G allele) conferring increased AD risk (*p* = 1.67 × 10^−11^; OR = 1.89; Figure 1A). LD analysis revealed that rs12555686 is in strong LD (r^2^ > 0.75) with two additional SNPs, rs2231225 and rs3758279. All three variants lie within 2 kb upstream of the *AQP3* transcription start site (NM_004925.5) and are predicted on SNPinfo (FuncPred) to overlap transcription factor binding motifs (Figure 1B). Haplotype analysis also showed that the G‐G‐G haplotype (rs12555686, rs2231225, rs3758279) was enriched in AD cases (*p* = 2.32 × 10^−7^; Figure 1B). eQTL analysis using GTEx v10 showed that individuals homozygous for the rs12555686 G allele exhibited significantly lower *AQP3* expression in skin (Figure 1C), whereas eQTLGen Phase II data confirmed the same association in whole blood (*p* = 9.48 × 10^−12^). These findings suggest that promoter variants have multitissue regulatory effects on *AQP3* expression.

**FIGURE 2:**
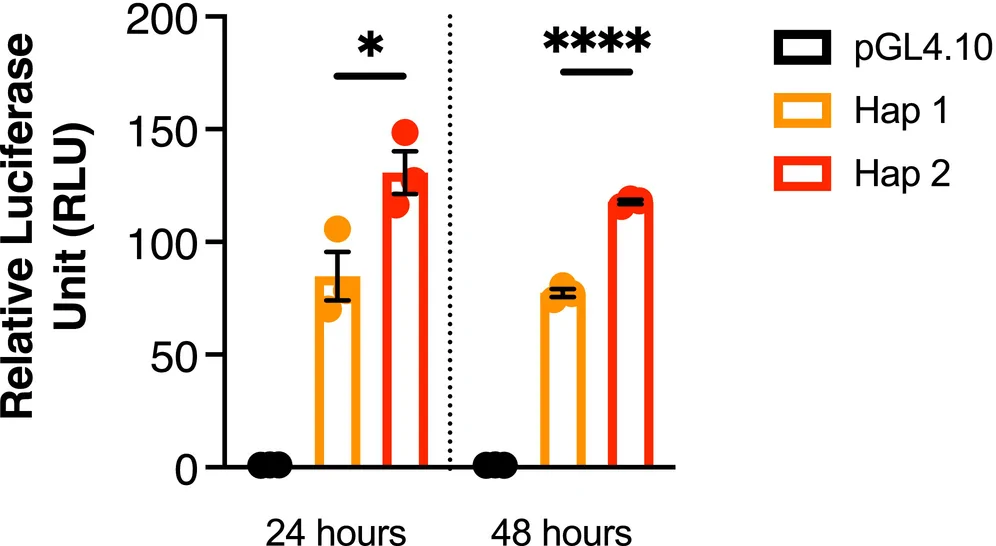
Relative luciferase unit (RLU) of luciferase activity in HEK293T cells transfected with either *AQP3* haplotype 1 (G‐G‐G) or 2 (T–T‐A). Haplotypes were cloned into the 5′UTR of pGL4.10 luciferase gene and measured at 24 or 48 h post‐transfection. Empty promoter‐less pGL4.10 included as background control. Data are mean ± SEM of three independent experiments with quadruplicate readings. Haplotypes 1 (Hap 1) and 2 (Hap 2) were analysed by two‐tailed Student's t test at 24 h and 48 h post‐transfection with mean RLU differences of 45.93 ± 14.34 (95% CI: 6.13 to 85.73; *p = 0.0328) and 40.32 ± 2.06 (95% CI: 34.60 to 46.05; ****p < 0.0001) respectively.

Consistently, luciferase reporter assays in HEK293T cells demonstrated reduced transcriptional activity of the G‐G‐G haplotype 1 (rs12555686, rs2231225, rs3758279) relative to the non‐risk haplotype 2 (T–T‐A) at 24 and 48 h post‐transfection (*p* < 0.05; Figure 2).

### 3.2 Reduced *AQP3* Expression Is Associated With Reduced Expression of Autophagy‐Related Genes and Increased Senescence‐Associated Gene Expression

To investigate whether *AQP3* haplotypes are associated with transcript expression levels of downstream autophagy‐ and senescence‐related genes, we retrieved ex vivo transcriptomic data of PBMCs from 658 participants from the SMCGES sub‐cohort (mean age = 22.99 ± 7.87, 32.4% male). The cohort comprised 112 AD and 546 non‐AD individuals. Detailed characteristics of this sub‐cohort have been reported previously [17].

**FIGURE 3:**
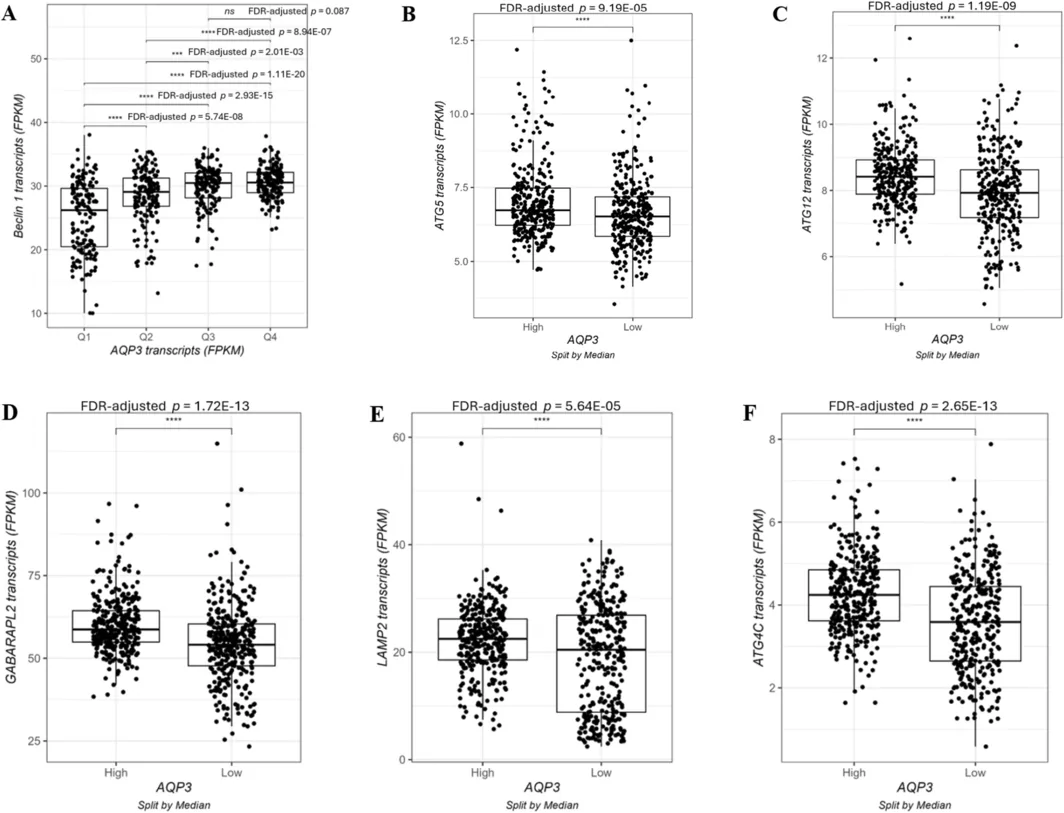
Expression of autophagy‐related genes is associated with *AQP3* transcript levels in PBMCs. (A) *Beclin 1*, (B) *ATG5*, (C) *ATG12*, (D) *GABARAPL2*, (E) *LAMP2*, and (F) *ATG4C* transcript levels (FPKM) are plotted against *AQP3* expression. In panel A, samples were stratified into quartiles (Q1–Q4) based on *AQP3* expression. In panels B–F, samples were split into High and Low groups relative to the median *AQP3* expression. Each dot represents an individual sample; box‐and‐whisker plots show the interquartile range and distribution. All analyses were performed on ex vivo transcriptomic data of PBMCs from 658 participants from the SMCGES sub‐cohort. Differences in gene expression between groups were compared using two‐tailed Mann–Whitney U tests, and the Benjamini‐Hochberg procedure was applied to calculate the false discovery rate (FDR). *p < 0.05, **p < 0.01, ***p < 0.001, and ****p < 0.0001.

Ex vivo transcriptomic analysis of PBMCs demonstrated that *AQP3* expression was significantly and positively correlated with canonical autophagy‐related genes, including *BECN1*, *ATG5*, *ATG12*, *GABARAPL2*, *LAMP2*, and *ATG4C* (*p* < 0.0001; Figure 3). Moreover, *BECN1* transcript levels increased progressively across quartiles of *AQP3* expression (*p* < 0.05; Figure 3A), supporting a dose‐dependent relationship. These findings are consistent with prior evidence that AQP3 promotes Beclin 1–mediated autophagy [3, 4, 5].

To explore whether these transcriptomic associations were also observed in skin tissue, we analysed a publicly available single‐cell RNA‐sequencing dataset of lesional skin samples from AD and psoriasis patients (GEO: GSE313454, 87 103 keratinocytes from 20 donors: 8 ad and 12 psoriasis). At the individual cell level, higher *AQP3* expression was significantly and positively associated with the expression of multiple autophagy‐related genes, including *BECN1, ATG5, GABARAPL2*, and *LAMP2* (Figure S1). As this analysis was performed at the single‐cell level without accounting for donor‐level correlation, these findings should be considered exploratory and interpreted with caution. Nevertheless, they provide supportive evidence that the positive associations between *AQP3* expression and autophagy‐related gene signatures are also observable in skin tissue.

**FIGURE 4:**
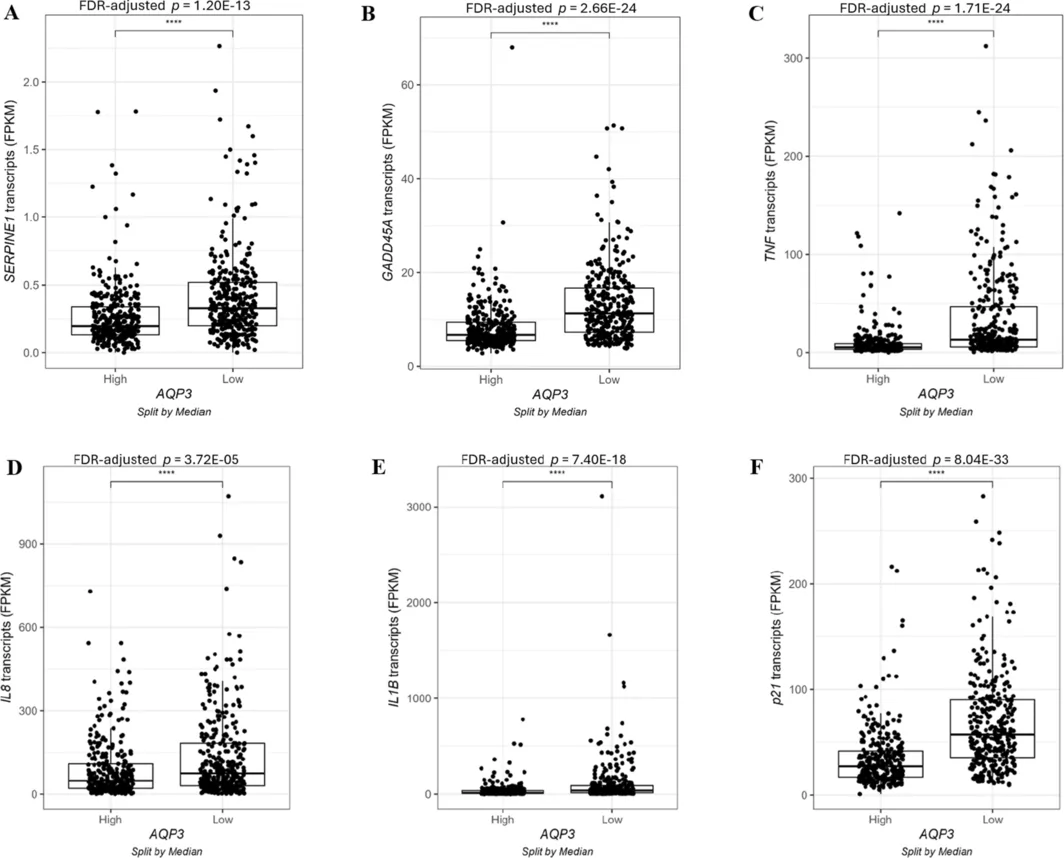
Expression of senescence‐associated genes in human PBMCs stratified by *AQP3* expression. Box and whisker plots display transcript levels (FPKM) of (A) *SERPINE1*, (B) *GADD45A*, (C) *TNF*, (D) *IL8*, (E) *IL1B* and (F) *p21* in human peripheral blood mononuclear cells (PBMCs) categorized into high and low *AQP3* expression groups (split by median). Each dot represents an individual sample. All analyses were performed on ex vivo transcriptomic data of PBMCs from 658 participants from the SMCGES sub‐cohort. Differences in gene expression between groups were compared using two‐tailed Mann–Whitney U tests, and the Benjamini‐Hochberg procedure was applied to calculate the false discovery rate (FDR). Boxes represent the interquartile range, with whiskers extending to 1.5× IQR. ****p < 0.0001.

Conversely, lower *AQP3* expression was associated with elevated expression of senescence and senescence‐associated secretory phenotype (SASP)‐related markers, including *SERPINE1*, *GADD45A*, *CDKN1A* (*p21*), *IL8*, *TNF*, and *IL1B* (*p* < 0.0001; Figure 4). Overall, these findings demonstrate an association between reduced *AQP3* expression, lower expression of autophagy‐related genes, and increased expression of senescence‐associated genes.

**FIGURE 5:**
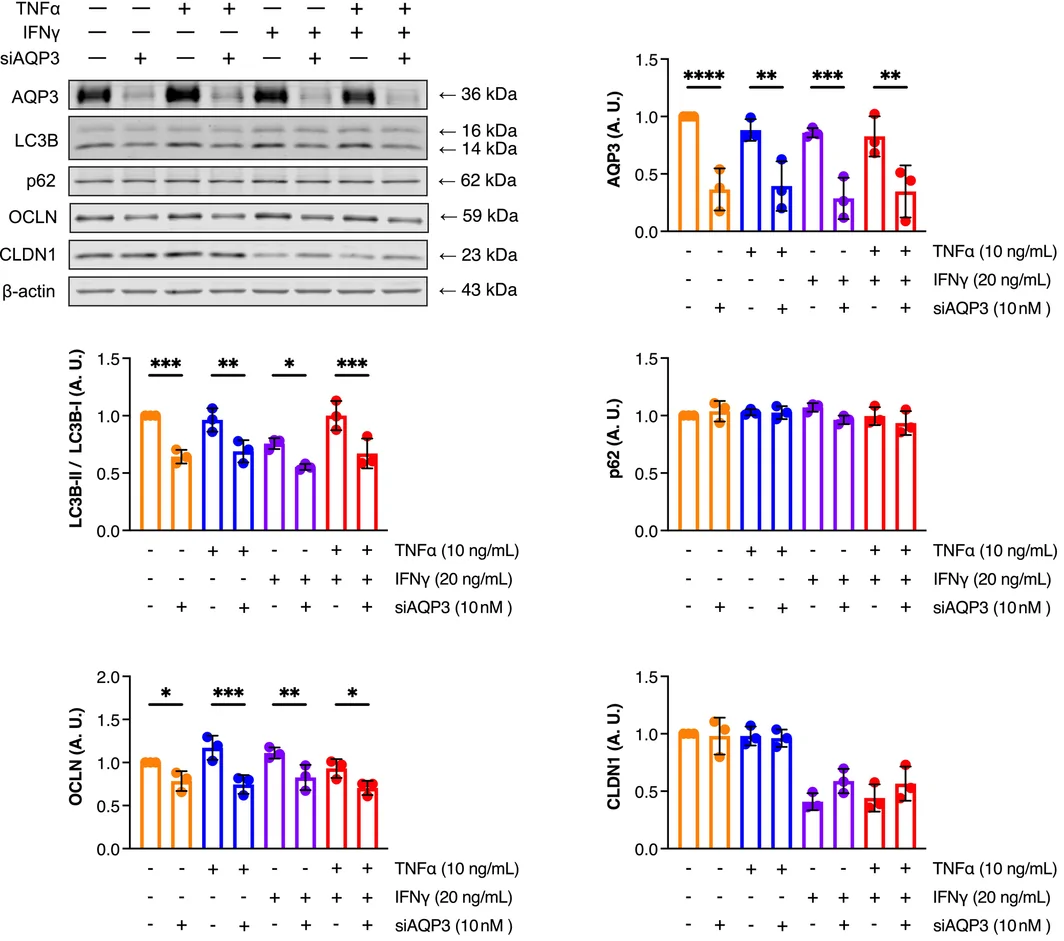
*AQP3* Knockdown reduces steady‐state LC3B‐II/LC3B‐I ratio and reduces occludin expression in HaCaT cells. Representative Western blots and corresponding bar graphs of AQP3, LC3B, p62, OCLN and CLDN1 with β‐Actin as loading control in HaCaT human keratinocyte cells with and without siRNA‐mediated *AQP3* knockdown (siAQP3) and stimulated with TNFα (10 ng/mL) and/or IFNγ (20 ng/mL). Data are mean ± SD of three independent experiments and analysed by two‐way ANOVA with Tukey's post hoc test to compare groups with and without siAQP3. Respective mean differences: AQP3, 0.54 ± 0.06 (95% Ci: 0.41 To 0.68; ****p < 0.0001); LC3B‐II/LC3B‐I, 0.29 ± 0.04 (95% CI: 0.22 To 0.37; ****p < 0.0001); OCLN, 0.29 ± 0.04 (95% CI: 0.20 To 0.38; ****p < 0.0001). A.U.: Arbitrary units, *p < 0.05, **p < 0.01, ***p < 0.001 and ****p < 0.0001.

To investigate the potential role of *AQP3* in AD skin, we suppressed *AQP3* expression in HaCaT keratinocytes using siRNA‐mediated knockdown. Reduced *AQP3* expression resulted in reduced steady‐state LC3B‐II/LC3B‐I ratios and reduced occludin expression under both basal and cytokine‐stimulated conditions (TNFα and IFNγ) (Figure 5). These findings suggest that *AQP3* may play a conserved role in regulating autophagy‐associated molecular readouts in both immune cells and skin keratinocytes.

### 3.3 *AQP3* Deficiency Impairs Wound Repair

**FIGURE 6:**
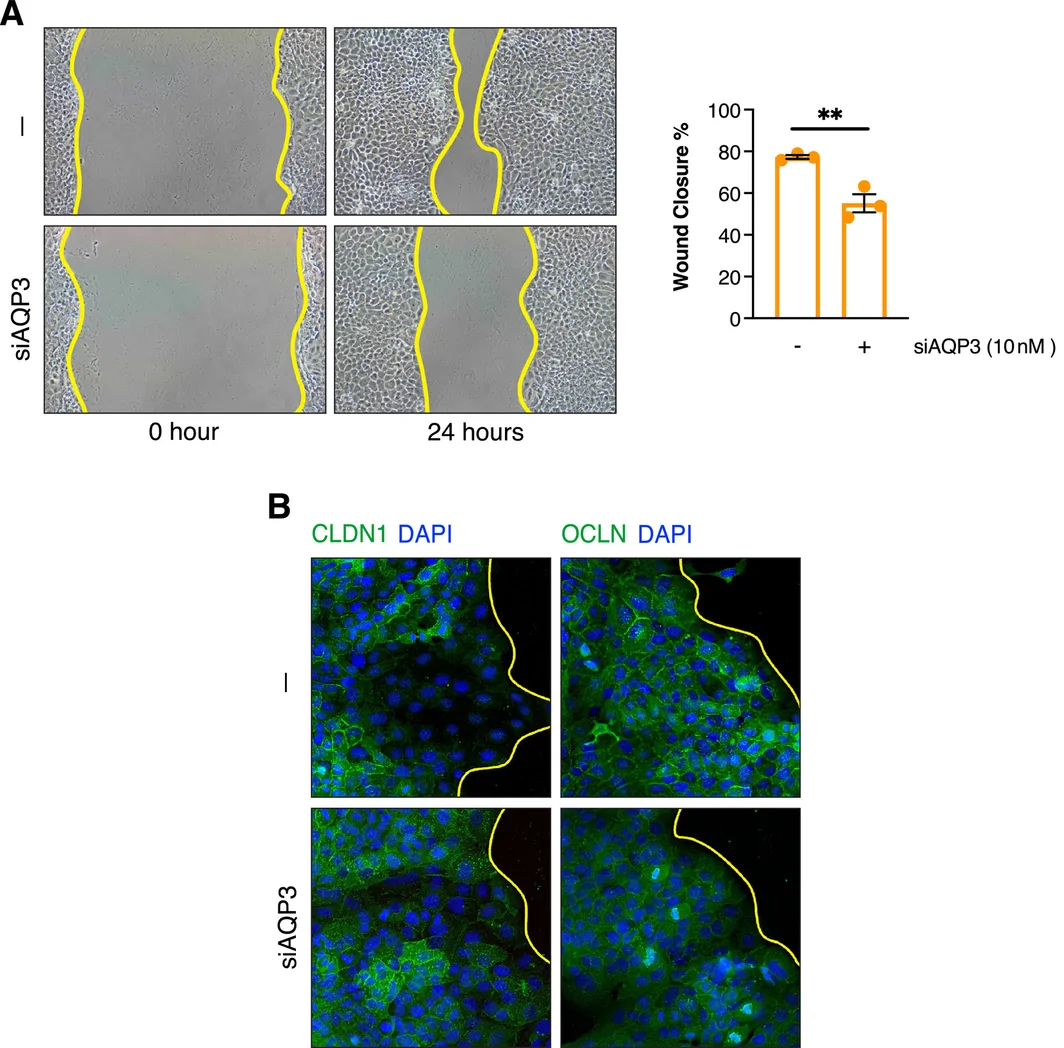
*AQP3* knockdown impairs wound closure in HaCaT keratinocytes. (A) Representative phase contrast microscopy images (100 × total magnification) and wound closure percentages of HaCaT keratinocytes with and without siRNA‐mediated *AQP3* knockdown (siAQP3) in a scratch wound assay after 24 h. Yellow outlines indicate cell‐free regions in HaCaT monolayers induced by the scratch at 0 and 24 h. Data are mean ± SEM of three independent experiments and analysed by two‐tailed Student's *t*‐test with a mean difference of −22.15% ± 4.43% (95% CI: −34.46 to −9.85; **p = 0.0075). (B) Representative immunocytochemistry (200 × total magnification) of scratch wound boundaries of HaCaT keratinocytes with and without siRNA‐mediated *AQP3* knockdown (siAQP3) for CLDN1 (green) or OCLN (green) with nuclei counterstain (DAPI, blue). Yellow outlines indicate the migratory front of HaCaT keratinocytes induced by the scratch after 24 h. Immunocytochemistry was repeated twice with consistent results.

A scratch wound assay was performed to assess the functional consequence of reduced *AQP3* expression and the associated reduced occludin expression in HaCaT keratinocytes (Figure 5). Wound closure was delayed upon *AQP3* knockdown, suggesting that *AQP3* may participate in regulating wound healing and maintaining skin homeostasis (Figure 6A). Immunocytochemistry staining of the wound edges revealed similar claudin 1 (CLDN1) expression and distribution between control and siAQP3 wounds. In contrast, occludin (OCLN) expression was diminished in siAQP3 wounds compared to control wounds (Figure 6B). Of note, both CLDN1 and OCLN have been shown to play important roles in wound healing [18, 19, 20].

## 4 Discussion

In this study, we identified a promoter haplotype in *AQP3* comprising rs12555686, rs2231225, and rs3758279, which was identified in the discovery GWAS as strong AD‐associated signals in a Singapore–Malaysia Chinese cohort. By integrating genome‐wide association analysis, cis‐eQTL data, luciferase reporter assays, PBMC transcriptomic profiling, analyses of an independent AD and psoriasis skin transcriptomic dataset, and genetic perturbation in keratinocytes, our findings suggest that this risk haplotype is associated with reduced *AQP3* transcription and altered expression of autophagy‐related genes relevant to AD risk and pathogenesis.

*AQP3* is a multifunctional water and glycerol channel expressed abundantly in keratinocytes and epithelial cells. Although originally characterized for its role in maintaining hydration and barrier integrity, *AQP3* is increasingly recognized as a dynamic modulator of cellular signalling, proliferation, migration, and survival. *AQP3* dysregulation has been implicated in several pathological conditions including skin cancer and psoriasis [21, 22]. Genetics studies have also linked *AQP3* locus variation to diverse diseases such as lung adenocarcinoma, Epstein–Barr virus–associated cancers, COVID‐19 severity, hypertensive disorders of pregnancy, and schizophrenia [23, 24, 25, 26]. These diverse associations underscore the regulatory sensitivity and functional plasticity of *AQP3*. Our results now extend these observations to AD, demonstrating that *AQP3* promoter haplotype (rs12555686, rs2231225, and rs3758279) is associated with differential transcriptional activity and contributes to disease‐relevant phenotypes. Moreover, the observed MAF differences of the tag‐SNP rs12555686 (allele T) among East Asian (0.141), European (0.06), American (0.163), and African (0.011) in the 1000 Genome database, and the present SMCGES cohort (0.094) suggest that the contribution of this haplotype to AD susceptibility may vary across populations.

Beyond its transport activity, *AQP3* also exerts signalling functions. It can regulate autophagy through direct binding to Beclin 1 or by releasing Beclin 1 from DEDD, thereby facilitating autophagosome formation [3, 4, 5]. This is particularly relevant in AD, where efficient autophagy is essential for removing damaged proteins, suppressing inflammation, and maintaining epidermal homeostasis. Our transcriptomic analysis revealed that lower *AQP3* expression in peripheral immune cells correlates with reduced expression of canonical autophagy‐related genes—such as *BECN1*, *ATG5*, and *GABARAPL2* (Figure 3) and a concomitant increase in senescence‐related markers, including *p21*, *SERPINE1*, *IL1B*, and *IL8* (Figure 4). These transcriptional shifts suggest that *AQP3* insufficiency may skew immune cells toward a pro‐inflammatory, pro‐senescent state, as evidenced by impaired autophagy observed in lesional skin and chronic inflammation in AD [15, 27]. Notably, PBMC gene signatures can only provide limited inference to AD skin biology due to gene regulation differences between cell types.

To investigate these findings in an epithelial context, we performed a luciferase promoter assay in HEK293T cells because of their high transfection efficiency and found that the risk haplotype 1 exhibited lower promoter activity than the non‐risk haplotype 2 (Figure 2). These findings suggest that *AQP3* promoter activity may be conserved between PBMCs and epithelial cells. However, *AQP3* expression has been reported to vary according to both cell type and the differentiation state of primary keratinocytes [28, 29]. Nevertheless, the reduced promoter activity of the rs12555686‐G risk haplotype 1 in HEK293T cells is consistent with the skin eQTL findings (Figure 1C and Figure 2).

*AQP3* was inhibited by siRNA‐mediated knockdown in HaCaT keratinocytes, resulting in reduced steady‐state LC3B‐II/LC3B‐I ratios and decreased occludin expression under both basal and cytokine‐stimulated conditions that mimic the inflammatory skin microenvironment [30, 31] (Figure 5). These findings are consistent with reports of *AQP3* downregulation and impaired autophagy in lesional skin [31, 32], although increased *AQP3* expression has also been associated with psoriasis severity [33, 34]. Similarly, *AQP3* downregulation has similarly been shown to reduce steady‐state LC3B‐II/LC3B‐I ratio in skin fibroblasts [5] and to compromise both autophagy and epithelial barrier integrity in intestinal cells [35, 36].

CLDN1 and OCLN are critical for skin regeneration, and OCLN knockdown impedes cell migration and wound closure in epithelial cell lines, including HaCaT keratinocytes [19, 37]. CLDN1 has been reported to be downregulated, whereas OCLN is upregulated, following injury in HaCaT keratinocytes [19], consistent with our observations at the migratory front of wound edges in control HaCaT keratinocytes following scratch injury (Figure 6B). Hence, we propose that the delayed wound closure observed in siAQP3 HaCaT keratinocytes may be attributable, at least in part, to reduced OCLN expression (Figure 5 and Figure 6). These findings support a role for *AQP3* in regulating skin regenerative responses, potentially through autophagy modulation and skin barrier maintenance, in addition to its canonical role as a water and glycerol transport channel.

AQP3 expression has been reported to be reduced in edematous regions of AD skin biopsies, as detected by immunostaining [38]. In contrast, increased AQP3 expression has also been observed in both non‐lesional and lesional AD skin biopsies compared with healthy skin, as determined by immunostaining and corroborated by *AQP3* transcript measurements [39, 40]. Although our experimental data focused on genetic perturbation, prior studies have reported that *AQP3* expression is highly responsive to environmental stressors, including UV radiation. For instance, acute UVA exposure can enhance *AQP3* transcription via JUN activation [5], whereas chronic UV exposure suppresses it through ROS‐induced MAPK signalling [41]. These dynamic, context‐dependent regulatory effects may help reconcile discrepancies in the literature regarding *AQP3* expression in AD [42]. Variability in UV exposure or genotype–environment interactions could contribute to these divergent patterns and may also explain the population‐specific nature of our association findings.

This study has several limitations. First, the association between *AQP3* promoter variants and AD was identified in a single discovery cohort and therefore requires validation in independent populations. To assess external support for this locus, we examined publicly available GWAS datasets, including BioBank Japan and the GWASCatalogue. However, the associations between AD and variants within the functional *AQP3* haplotype (rs12555686, rs2231225, and rs3758279) were not consistent with the findings of the present study (Table S1). This discrepancy may reflect differences in ancestry‐specific allele frequencies, linkage disequilibrium patterns, environmental exposures, statistical power, or genetic architecture between populations. Consequently, the generalizability of the observed association remains uncertain. Future studies involving larger independent cohorts, particularly across multiple ancestries, together with fine‐mapping and functional validation approaches, will be required to further elucidate the role of *AQP3* regulatory variants in AD susceptibility. Second, the exploratory single‐cell RNA‐sequencing analysis was performed at the individual cell level using an independent AD and psoriasis skin transcriptomic dataset and did not account for donor‐level correlation through pseudobulk or mixed‐effects approaches (Figure S1). Consequently, these findings should be regarded as supportive, hypothesis‐generating evidence rather than independent validation of the PBMC transcriptomic associations. Future studies using appropriately powered donor‐level analyses will be important to confirm these observations.

**FIGURE 7:**
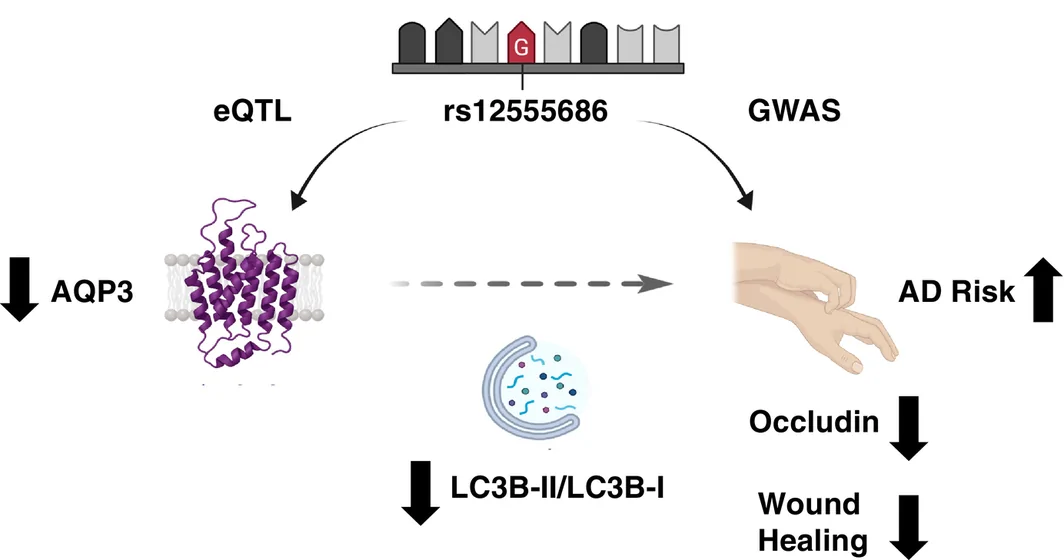
Proposed hypothesis of *AQP3* rs12555686's influence on AD. The major allele “G” of rs12555686 located in the *AQP3* promoter region was associated with lower *AQP3* expression and increased AD risk through expression quantitative trait loci (eQTL) analyses and genome‐wide association study (GWAS) respectively. Based on the in vitro knockdown of *AQP3* in HaCaT keratinocytes, we propose that reduced *AQP3* expression in atopic dermatitis (AD) skin contributes to disease pathogenesis by reduced steady‐state LC3B‐II/LC3B‐I ratio, reduced occludin expression, and delayed wound healing. Figure created with BioRender.com.

In summary, we propose that the rs12555686‐G risk promoter haplotype reduces *AQP3* gene expression, thereby contributing to reduced steady‐state LC3B‐II/LC3B‐I ratio, reduced occludin expression, and delayed wound healing, all of which are characteristic features of AD pathology (Figure 7). Understanding this regulatory axis could offer new mechanistic insight and potentially therapeutic avenues in AD and other inflammatory skin disorders.

## Author Contributions

F.T.C. conceived and supervised the overall research study. Y.Y.E.L. planned, conducted the experiments, and analysed the data. Y.Y.E.L. wrote the manuscript. Y.Y.S. assisted in collecting and consolidating the data. T.Y.W.L. conducted experiments and analysed the data during manuscript revision. Y.‐H.S., and K.R. assisted in recruiting participants. All authors reviewed, revised, and approved the final manuscript.

## Funding

This work was supported by National University of Singapore, N‐154‐000‐038‐001 (E‐154‐00‐0017‐01), C141‐000‐077‐001 (E‐141‐00‐0096‐01). Singapore Ministry of Education Academic Research Fund, R‐154‐000‐191‐112, R‐154‐000‐404‐112, R‐154‐000‐553‐112, R‐154‐000‐565‐112, R‐154‐000‐630‐112, R‐154‐000‐A08‐592, R‐154‐000‐A27‐597, R‐154‐000‐A91‐592, R‐154‐000‐A95‐592, R154‐000‐B99‐114. Biomedical Research Council, BMRC/01/1/21/18/077, BMRC/04/1/21/19/315, BMRC/APG2013/108. Singapore Immunology Network, SIgN‐06‐006, SIgN‐08‐020. National Medical Research Council, NMRC/1150/2008, OFIRG20nov‐0033, MOH‐001636, (OFLCG23may‐0038, A‐8002641‐00‐00). National Research Foundation Singapore, NRF‐MP‐2020‐0004. Singapore Food Agency, SFS_RND_SUFP_001_04, W22W3D0006, NRF‐SFSRND2SIH‐0001, SFS_RND_2_FS_0002. Singapore's Economic Development Board, A‐8002576‐00‐00. Agency for Science, Technology and Research, H17/01/a0/008, APG2013/108.

## Ethics Statement

Study subjects residing in Singapore or Malaysia were recruited from the National University of Singapore (NUS), Sunway University or Universiti Tunku Abdul Rahman (UTAR) through various ongoing cross‐sectional volunteer recruitment drives. Approvals were obtained from the Institutional Review Board of NUS (IRB, Reference‐NUS‐07‐023, NUS‐09‐256, NUS‐10‐445, NUS‐13‐075, NUS‐14‐150, and NUS‐18‐036), the Scientific and Ethical Review Committee of UTAR (Reference‐U/SERC/03/2016), and the Sunway University Research Ethics Committee (Reference‐SUREC 2019/029), and in compliance with the Helsinki Declaration. Written informed consent was also obtained on site via a Patient Information Sheet (PIS) for those aged 18 and above. Those under 21 years of age at time of participation were also required to submit parental or guardian consent. A subset of participants recruited for the cross‐sectional study provided whole blood samples, collected by certified phlebotomists or nurses, with written informed consent and approval from the Scientific and Ethical Review Committee (SERC) of UTAR (Ref‐code: U/SERC/03/2016), Sunway University Research Ethics Committee (Ref‐SUREC 2019/029), and the Institutional Review Board at the National University of Singapore, Singapore (IRB numbers: NUS 07‐023, NUS 09‐256, and NUS 10‐445).

## Conflicts of Interest

F.T.C reports grants from the National University of Singapore, Singapore Ministry of Education Academic Research Fund, Singapore Immunology Network, National Medical Research Council (NMRC) (Singapore), Biomedical Research Council (BMRC) (Singapore), National Research Foundation (NRF) (Singapore), Singapore Food Agency (SFA), Singapore’s Economic Development Board (EDB), and the Agency for Science Technology and Research (A*STAR) (Singapore), during the conduct of the study; and consulting fees from Sime Darby Technology Centre; First Resources Ltd; Genting Plantation, Olam International, Musim Mas, and Syngenta Crop Protection, outside the submitted work. The other authors declare no other competing interests.

## Supporting information


**Figure S1:** Exploratory cell‐level co‐expression of *AQP3* and autophagy‐related genes in skin keratinocytes (GEO: GSE313454).
**Figure S2:** QQ plot of the discovery genome‐wide association study (GWAS) of atopic dermatitis (AD) in the SMCGES cohort, which comprises 1261 ad cases and 4062 controls.
**Table S1:** Association of genetic variants in the AQP3 functional haplotypes with atopic dermatitis (AD) in public database.

## Data Availability

The data that support the findings of this study are available from the corresponding author upon reasonable request.
