# Supplementary material for: Aquaporin 3 Promoter Polymorphisms Reduce Gene Expression and Associate Altered Autophagy‐Related Gene Signatures With Atopic Dermatitis Susceptibility
Source: Exp Dermatol. 2026 Sep 15;35(9):e70358. doi: 10.1111/exd.70358 (PMC13577822; doi:10.1111/exd.70358)
Supplement: Supplementary file 1 [file EXD-35-e70358-s001.docx]

**Figure S1.** Exploratory cell-level co-expression of *AQP3* and autophagy-related genes in skin keratinocytes (GEO: GSE313454).

(A) *BECN1* (*Beclin 1*), (B) *ATG5*, (C) *GABARAPL2*, and (D) *LAMP2* expression levels (normalized counts) in epidermal keratinocytes (*n* = 87,103 cells isolated across *n* = 20 total donors, including 8 atopic dermatitis and 12 psoriasis patients). All cells were stratified into three categories based on *AQP3* expression (normalized counts), including no expression (0 count), as well as high and low expression groups relative to the median of overall *AQP3* expression counts. Box-and-whisker plots show the median and interquartile range and distribution. Statistical significance for pairwise comparisons was determined by Mann-Whitney U tests. *P*-values are denoted as: *p* < 0.0001 (****).

**Figure S2.** QQ plot of the discovery genome-wide association study (GWAS) of atopic dermatitis (AD) in the SMCGES cohort, which comprises 1,261 AD cases and 4,062 controls.

**Table S1**. Association of genetic variants in the AQP3 functional haplotypes with atopic dermatitis (AD) in public database.

| Proxy SNP | Assessed allele | Reference allele | P-value | Effect Size (SE) | Phenotype | Sample Size  (*N*) | Database |
| --- | --- | --- | --- | --- | --- | --- | --- |
| rs12555686 | T | G | 0.11 | 0.075 (0.046) | Atopic Dermatitis | 168,103 East Asian | BioBank Japan |
| rs12555686 | T | G | 0.47 | -0.128 (0.176) | Dermatitis and eczema | 2,198 East Asian | GWAS Catalog  (accession: GCST90473916) |
| rs2231225 | T | G | 0.0022* | 0.045 (0.015) | Atopic Dermatitis | 882,024 European  212,036 East Asian | GWAS Catalog  (accession: GCST90297795) |

SE, standard error. GWAS results were retrieved from Biobank Japan (https://pheweb.jp/) and GWAS Catalog (https://www.ebi.ac.uk/gwas/).
